# Abnormal interneuron development in *disrupted-in-schizophrenia-1* L100P mutant mice

**DOI:** 10.1186/1756-6606-6-20

**Published:** 2013-04-30

**Authors:** Frankie HF Lee, Clement C Zai, Sabine P Cordes, John C Roder, Albert HC Wong

**Affiliations:** 1 Campbell Family Mental Health Research Institute, Centre for Addiction and Mental Health, 250 College Street, Toronto, ON, M5T 1R8, Canada; 2 Department of Pharmacology, University of Toronto, Toronto, ON, M5S 1A8, Canada; 3 Department of Molecular Genetics, University of Toronto, Toronto, ON, M5S 1A8, Canada; 4 Samuel Lunenfeld Research Institute, Mount Sinai Hospital, Toronto, ON, M5G 1X5, Canada; 5 Department of Psychiatry, University of Toronto, Toronto, ON, M5T 1R8, Canada; 6 Institute of Medical Sciences, University of Toronto, Toronto, ON, M5S 1A8, Canada

**Keywords:** Disrupted-in-Schizophrenia 1 (DISC1), Interneuron, Mutant mouse, Schizophrenia

## Abstract

**Background:**

Interneuron deficits are one of the most consistent findings in post-mortem studies of schizophrenia patients and are likely important in the cognitive deficits associated with schizophrenia. Disrupted-in-Schizophrenia 1 (DISC1), a strong susceptibility gene for schizophrenia and other mental illnesses, is involved in neurodevelopment, including that of interneurons. However, the mechanism by which DISC1 regulates interneuron development remains unknown. In this study, we analyzed interneuron histology in the *Disc1*-L100P single point mutation mouse, that was previously shown to have behavioral abnormalities and cortical developmental defects related to schizophrenia.

**Results:**

We sought to determine whether a *Disc1*-L100P point mutation in the mouse would alter interneuron density and location. First, we examined interneuron position in the developing mouse cortex during embryonic days 14–16 as an indicator of interneuron tangential migration, and found striking migration deficits in *Disc1*-L100P mutants. Further analysis of adult brains revealed that the *Disc1*-L100P mutants have selective alterations of calbindin- and parvalbumin-expressing interneurons in the cortex and hippocampus, decreased GAD67/PV co-localization and mis-positioned interneurons across the neocortex when compared to wild-type littermates.

**Conclusion:**

Our results are consistent with the anomalies seen in post-mortem schizophrenia studies and other *Disc1* mutant mouse models. Future research is required to determine the specific mechanisms underlying these cellular deficits. Overall, these findings provide further evidence that DISC1 participates in interneuron development and add to our understanding of how *DISC1* variants can affect susceptibility to psychiatric illness.

## Background

Cognitive control depends on neural synchrony that maintains a balanced excitation and inhibition in different brain regions [1]. GABAergic interneurons are critical for providing inhibitory control over pyramidal neurons and modulating synchronized oscillations [2]. Interneuron deficits have been one of the most consistent findings in human post-mortem schizophrenia studies, including reductions in glutamic acid decarboxylase-67 (GAD67) expression, and parvalbumin (PV) mRNA expression and immunoreactivity [3-5]. Different interneuron subtypes have distinct electrophysiological and synaptic characteristics [6]. In schizophrenia, GAD67 reduction appears to be restricted to PV-interneurons [7,8]. This is of particular relevance as recent optogenetic studies on animal models have shown that PV-interneurons are required for generating gamma-frequency oscillations [9,10], that are critical for cognition [11,12]. Consistent with this notion, schizophrenia patients display abnormal neural oscillations and synchronizations [13,14]. Furthermore, rodents with loss of PV-interneurons and impaired gamma activity show selective cognitive deficits reminiscent of schizophrenia symptoms [15,16].

Disrupted-in-schizophrenia 1 (*DISC1*) is a strong susceptibility gene for schizophrenia and other mental disorders [17]. DISC1 functions as a scaffold protein and regulates a wide-range of neurodevelopmental processes [18-20]. Different mutant DISC1 mouse models have displayed selective reductions in PV interneurons [21-24] and alterations in their laminar distribution [22]. Recently, Steinecke *et al.* demonstrated that DISC1 also regulates interneuron tangential migration [25], further supporting a possible role for DISC1 in modulating interneuron development.

Our group previously described a mutant mouse line, *Disc1*-L100P that has behavioral and cognitive abnormalities related to schizophrenia [26], consistent with four other publications [27]. Given the accumulating evidence for DISC1 and interneuron abnormalities in schizophrenia, we undertook a comprehensive histological analysis of interneurons in the *Disc1*-L100P mutants. Our findings suggest that *Disc1* mutations may have distinct spatial and temporal effects in different interneuron subtypes. Overall, our study provides evidence for the effects of *Disc1* SNPs on interneuron development that represent a starting point for further investigations into developmental and pathophysiological mechanisms in schizophrenia.

## Results

### Impaired tangential interneuron position in the embryonic *Disc1*-L100P mouse

**Figure 1:**
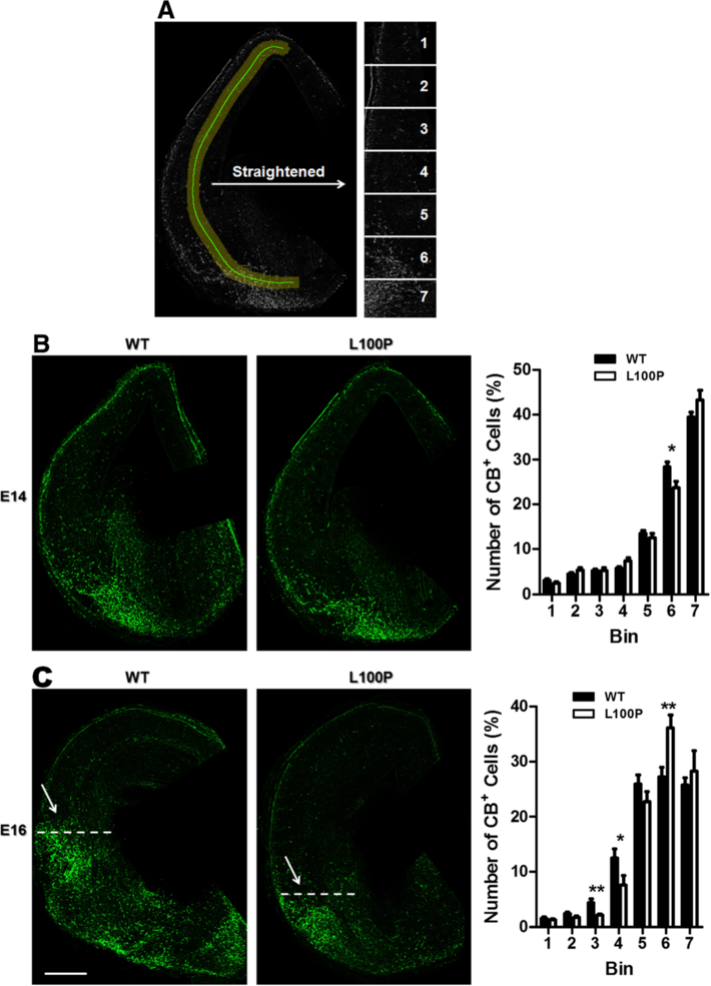
**Disrupted interneuron tangential migration in *Disc1-*L100P embryonic mice.** (**A**) Coronal sections of either E14 or E16 embryonic brains immunostained with CB were straightened and divided into seven equidistant bins covering the tangential migratory pathway of interneurons. Fluorescently-labeled cell numbers were counted in each bin and normalized to the total number in all bins. The distribution of these interneurons was significantly different for both E14 and E16 time points between WT and *Disc1*-L100P (two-way ANOVA, *p* < 0.01). (**B**) E14 CB-immunolabeled images of WT and *Disc1-*L100P mice are shown on the left. *Disc1-*L100P mice had a significantly lower proportion of CB-cells in the ventral bin 6 when compared to WT controls (n = 25–28 sections from 4 different embryos per group, *t*-test, *p* < 0.05). (**C**) At E16, a more pronounced difference in interneuron tangential migration was observed between WT and *Disc1-*L100P mice (white arrows). *Disc1-*L100P mutants had significantly less CB^+^ cells in bins 3 and 4, while more remained near the ventral bin 6 when compared to WT (n = 19–24 sections from 4 different embryos per group, *t*-test, *p* < 0.01). Scale Bar, 300 μm. All data are shown as mean ± SEM; * *p* < 0.05, ** *p* < 0.01 versus WT. CB, calbindin.

Recent evidence suggests that DISC1 is likely to play an important role in interneuron tangential migration [25,28]. Thus, we compared the tangential migratory pathways in embryonic mouse brains of wild-type (WT) and *Disc1*-L100P mutants at two different time points, E14 and E16 by immunostaining with an early interneuron marker, calbindin (CB) [29] (Figure 1A). At E14, *Disc1*-L100P mutants had a lower proportion of CB-cells in bin 6 (23.73 ± 1.46%) while slightly higher in bin 7 when compared to WT (bin 6: 28.47 ± 1.02%) (Figure 1B). But at E16, we observed significantly more CB-interneurons in the dorsal cortex of bins 3 and 4 in WT controls (bin 3: 4.44 ± 0.7%; bin 4: 12.56 ± 1.62%) when compared to the *Disc1*-L100P mutants (bin 3: 2.14 ± 0.24%; bin 4: 7.62 ± 1.69%), while a larger proportion remained near the ventral sites of bin 6 in the mutant mice (WT: 27.24 ± 1.75%; L100P: 36.14 ± 2.3%) (Figure 1C). This indicates that migrating interneurons in the *Disc1*-L100P mutants failed to reach their proper dorsal target position, suggesting that the L100P mutation in the *Disc1* gene is likely to disrupt the tangential migration of interneurons.

### Altered CB- and PV-expressing interneuron numbers in the mPFC and DLFC of *Disc1*-L100P mutant mice

**Figure 2:**
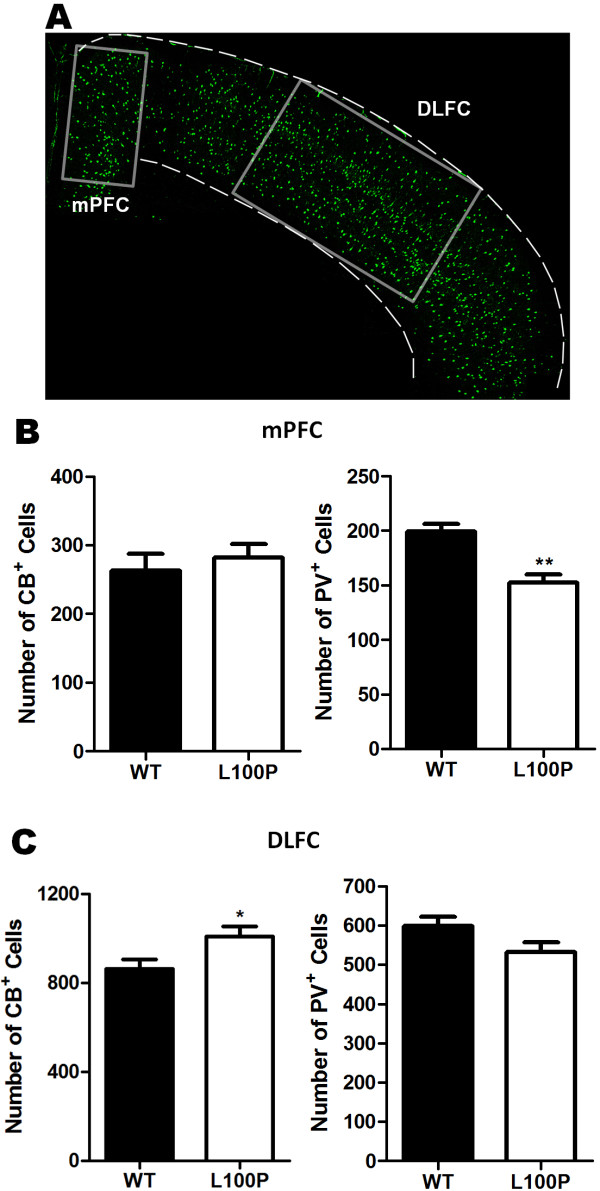
**Differential alterations in CB- and PV-labeled interneurons in the mPFC and DLFC of *Disc1*-L100P adult mice.** (**A**) A representative section used for counting CB- and PV-labeled cell densities is shown with boxes depicting the areas analyzed for the mPFC and DLFC regions. Scale Bar, 500 μm. (**B**) Quantification of CB^+^ and PV^+^ cells in the mPFC showed significantly fewer PV-interneurons in the *Disc1-*L100P mutants but no significant difference with CB-interneurons when compared to WT (n = 13–14 from 4 mice per group; *t*-test, *p* < 0.01). (**C**) In contrast, *Disc1-*L100P mutants have significantly more CB^+^ cells but not PV^+^ cells in the DLFC versus WT (n = 21–22 from 4 mice per group, *t*-test, *p* < 0.05). All data are shown as mean ± SEM; * *p* < 0.05, ** *p* < 0.01 versus WT. CB, calbindin; DLFC, dorsal lateral frontal cortex; mPFC, medial prefrontal cortex; PV, parvalbumin.

To address whether interneuron deficits remain as pronounced in our *Disc1*-L100P mouse model or become more diffuse as seen in post-mortem analyses, we examined the number of interneurons in both the medial prefrontal cortex (mPFC) and dorsolateral frontal cortex (DLFC) of the adult mouse (Figure 2A). Previous mutant DISC1 animal studies have demonstrated that interneuron deficits are present in these mouse brain regions [21-24]. Two different subclasses of interneurons (CB and PV) were immunolabeled with their respective antibodies, and labeled cells were counted in WT and *Disc1*-L100P mutants. In the mPFC, we observed significantly fewer PV-interneurons in L100P mutants (152.48 ± 7.46) when compared to WT (199.27 ± 7.28), consistent with previous reports [22]. However, there was no significant difference in CB-interneuron density (Figure 2B). Interestingly, L100P mutants had more CB-labeled cells (1008.31 ± 44.29) within the DLFC compared to WT (862.15 ± 43.06), while no alterations were observed with PV-labeled cells (Figure 2C). In addition, the average overall number of both interneuron subtypes was significantly higher in WT controls than the mutants within the mPFC, but there was no difference within the DLFC (Additional file 1: Figure S1). Our results show that *Disc1* mutations affect the numbers of each interneuron subtype differently.

### Aberrant interneuron laminar position in *Disc1*-L100P mutants

**Figure 3:**
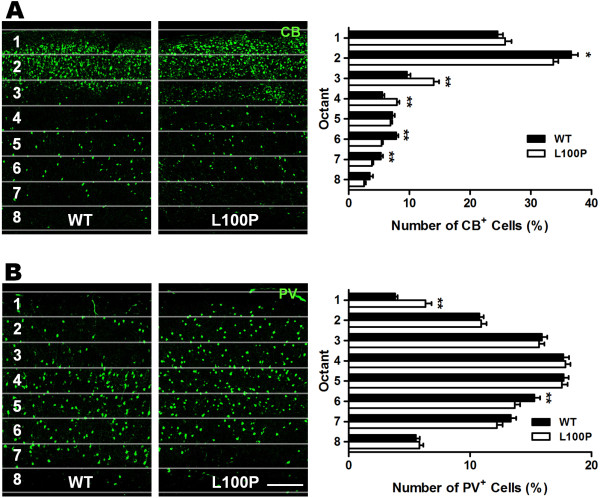
**Altered cortical interneuron laminar distribution in *Disc1-*L100P adult mice.** Quantification of both CB- and PV-labeled cells was achieved by counting the number of fluorescent cells in each octant and expressed as a percentage of total number per ROI of fixed size across the neocortex. (**A**) There was a significant interaction effect with CB-interneurons across the octants between WT and *Disc1-*L100P mice (two-way ANOVA, *p* < 0.01) with proportionally more CB-interneurons in the middle cortical layers of octants 3 and 4 (*t*-test, *p* < 0.01). Conversely, there were fewer CB^+^ cells in the second, sixth and seventh octants in the *Disc1-*L100P mutant versus WT (n = 52 ROIs from 4 different mice per group; *t*-test, *p* < 0.01). (**B**) PV-labeled interneurons were significantly different in their distribution across the octants between WT and *Disc1-*L100P mutants (two-way ANOVA, *p* < 0.01). More PV + cells were observed in WT of octant 6 but less in superficial cortical layers of octant 1 than in the mutants (n = 84–90 ROIs from 5 different mice per group; *t*-test, *p* < 0.01). Scale Bar, 300 μm. All data are shown as mean ± SEM; * *p* < 0.05, ** *p* < 0.01 versus WT. CB, calbindin; PV, parvalbumin; ROI, region of interest.

Post-mortem human and mutant DISC1 animal studies have reported abnormally located interneurons within cortical layers [8,22,30]. In our study, we investigated interneuron laminar position by analyzing CB- and PV-immunolabeled cells in a series of equally-spaced regions spanning the thickness of the cerebral cortex within the DLFC. We found an abnormal distribution of both interneuron subtypes in *Disc1*-L100P mice. The percentage of CB-interneurons was significantly higher in octants 3 and 4 of *Disc1*-L100P mutants (octant 3: 13.98 ± 0.91%; octant 4: 7.94 ± 0.43%) compared to WT (octant 3: 9.58 ± 0.58%; octant 4: 5.52 ± 0.34%), while WT controls exhibit a more dispersed pattern with higher percentages located in superficial and deeper cortical layers (Figure 3A). Conversely, the distribution pattern of PV-interneurons was similar between WT and *Disc1*-L100P mutants, but mutants displayed a shift towards more superficial cortical layers with a significantly higher proportion in octant 1 (6.32 ± 0.5%) and lower in octant 6 (13.68 ± 0.42%) versus WT controls (octant 1: 3.81 ± 0.22%; octant 6: 15.28 ± 0.47%) (Figure 3B). These data suggest an aberrant localization of cortical interneurons in *Disc1*-L100P mice that varies with each interneuron subtype.

### More PV-interneurons located in the lateral neocortex of *Disc1*-L100P mice

**Figure 4:**
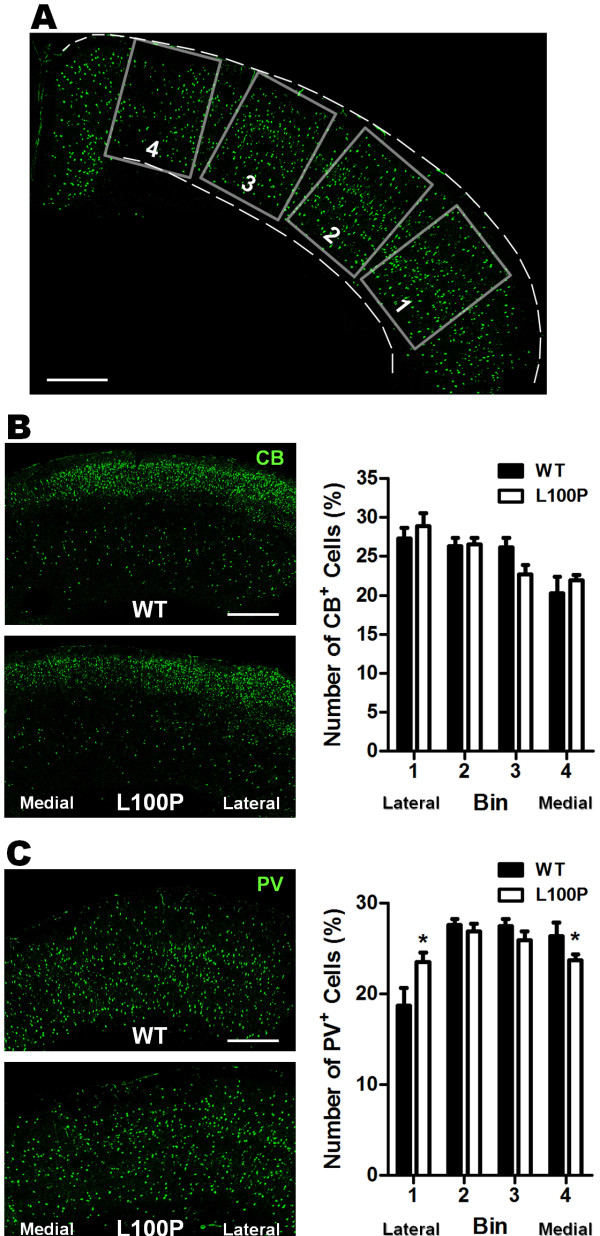
**Aberrant tangential distribution of PV-interneurons in *Disc1-*L100P adult mice.** (**A**) Four sampling ROIs immunolabeled with either CB or PV antibodies were outlined across the neocortex along the medial-lateral axis. The number of CB^+^ and PV^+^ cells were counted and expressed as a percentage of total cells in each ROI. (**B**) There was no significant difference in the tangential distribution of CB^+^ cells between WT and *Disc1-*L100P mice (n = 12–13 ROIs from 4 different mice per group). (**C**) PV-labeled cells were distributed differently depending on genotype. The *Disc1-*L100P mutants had a significantly higher proportion of PV^+^ cells located laterally (bin 1) but fewer PV^+^ cells in medial bin 4 when compared to WT (n = 22 ROIs from 4 different mice per group; two-way ANOVA, *t*-test, *p* < 0.05). Scale Bar, 500 μm. All data are shown as mean ± SEM; * *p* < 0.05 versus WT. CB, calbindin; PV, parvalbumin; ROI, region of interest.

Interneurons migrate from the ganglionic eminence of the telencephalon, moving from the lateral to medial cortex [31]. To further support our previous results of tangential migration deficits, we examined the distribution of interneurons across the medial-lateral axis of the neocortex in adult WT and *Disc1*-L100P mutant mice (Figure 4A). We found no substantial change in the distribution of CB-interneurons across the neocortex (Figure 4B). However for PV-interneurons in *Disc1*-L100P mutants, a higher proportion of fluorescent cells were situated in the lateral cortex and fewer reached the medial region when compared to WT controls (two-way ANOVA, Bin 1: WT – 18.67 ± 1.98%, L100P – 23.52 ± 1.02%; Bin 4: WT – 26.34 ± 1.51%, L100P – 23.7 ± 0.62%) (Figure 4C). These results indicate that the *Disc1*-L100P mutation only affected the tangential positioning of PV interneurons within the cortex, which may reflect a defect in interneuron tangential migration.

### Less GAD67/PV co-localization in *Disc1*-L100P mice

**Figure 5:**
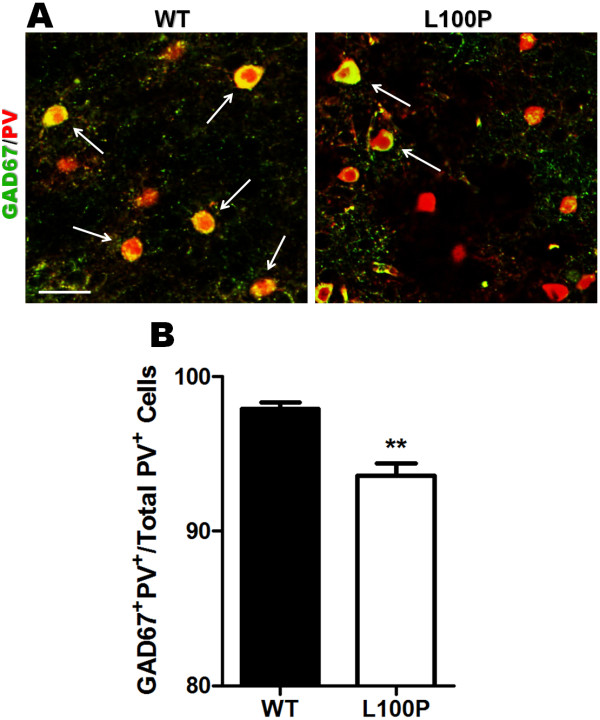
**Reduced GAD67/PV co-localization in *Disc1-*L100P adult mutants.** (**A**) GAD67 (green) and PV (red) fluorescently labeled images in WT and *Disc1-*L100P mutants. White arrows indicate those with more than 50% overlap of GAD67 and PV. Scale Bar, 50 μm. (**B**) Quantification of the percentage of GAD67^+^PV^+^ cells per total PV^+^ cells revealed significantly less GAD67 expression within PV-cells in *Disc1-*L100P mice compared to WT (n = 106–128 ROIs from 4 different mice per group). All data are shown as mean ± SEM; ** *p* < 0.01 versus WT. GAD67, glutamic acid decarboxylase (67 kDa); PV, parvalbumin.

One of the most robust findings in post-mortem schizophrenia studies is a reduction in GAD67 expression, preferentially within PV-interneurons [7,8]. However, there is still a lack of evidence for the effect of DISC1 on GAD67 expression. Hence we sought to determine the co-localization of GAD67 and PV in our *Disc1*-L100P mutants (Figure 5A). Consistent with previous reports, we found significantly fewer GAD67^+^PV^+^ cells in the *Disc1*-L100P mutants (93.57 ± 0.81%) vs. WT (97.93 ± 0.40%) (Figure 5B), indicating that DISC1 may affect GAD67 expression.

### Increased hippocampal PV-interneurons in *Disc1*- L100P mutants

**Figure 6:**
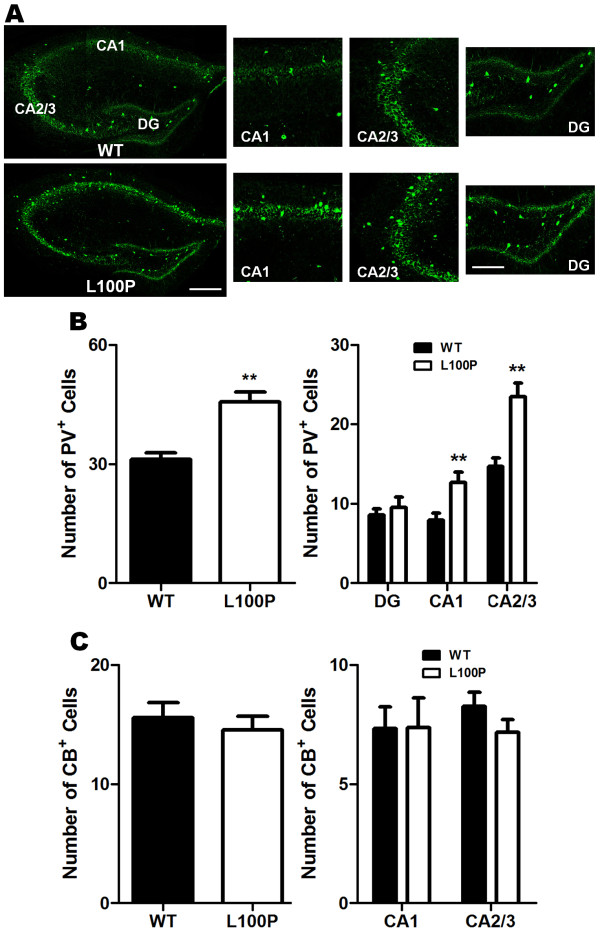
**Selective increase of PV-interneurons in CA1 and CA2/3 regions of the hippocampus in *Disc1-*L100P adult mutant mice.** (**A**) PV-immunostained images of the hippocampus in WT and *Disc1-*L100P mice were divided into CA1, CA2/3 and DG subfields in which labeled cells were counted. Scale Bar, 300 μm. A higher magnification of each subfield is shown on the right. Scale Bar, 100 μm. (**B**) *Disc1-*L100P mutants had significantly more PV-interneurons in the hippocampus, particularly within CA1 and CA2/3 subfields when compared to WT (n = 12 ROIs for each region from 4 different mice per group). (**C**) There was no significant difference in the number of CB^+^ interneurons within the CA1 and CA2/3 regions between WT and *Disc1-*L100P mutants. DG was excluded from analysis due to the absence of CB-interneurons. All data are shown as mean ± SEM; ** *p* < 0.01 versus WT. CB, calbindin; PV, parvalbumin; CA, Cornu Ammonis; DG, dentate gyrus.

There is evidence for hippocampal interneuron deficits in schizophrenia [32]. Thus, we measured both CB- and PV-interneuron density within all subfields of the hippocampus in *Disc1*-L100P mice (Figure 6A). Surprisingly, we observed significantly more PV^+^ cells within the hippocampus specifically in the CA1 and CA2/3 regions of the *Disc1*-L100P mutants (CA1: 12.64 ± 1.36; CA2/3: 23.45 ± 1.73) when compared to WT controls (CA1: 7.92 ± 0.87; CA2/3: 14.67 ± 1.1) (Figure 6B). CB-labeled cell numbers were not significantly different in all hippocampal subfields (Figure 6C). Our data are inconsistent with previous reports of fewer hippocampal PV-interneuron numbers in schizophrenia, but suggest that the *Disc1-*L100P mutation results in more PV^+^ cells.

## Discussion

There is substantial evidence for an association between *DISC1* and several major mental illnesses. However, the mechanism by which *DISC1* gene variants produce both cellular and behavioral abnormalities is still unclear. In this study, we examined embryonic interneuron tangential migratory position and adult histology of two interneuron subtypes (CB and PV) in a mouse with a point mutation in the *Disc1* gene (L100P), which has been previously shown to have behaviors relevant to schizophrenia [26].

A recent study suggests that DISC1 is necessary for proper tangential migration of cortical interneurons [25]. Therefore, we examined the tangential migratory pathway of interneurons at E14 and E16, as an indicator of migration. Consistent with the putative role for DISC1 in interneuron development, our study revealed that the *Disc1*-L100P mutants displayed abnormal tangential migration. This was further supported by our findings that PV-interneurons remained in the lateral adult cortex and that there were fewer interneurons overall in the mPFC. A plausible explanation is that the L100P mutation disrupts specific DISC1 protein interactions and results in mis-regulated downstream signals. ErbB4, and its substrate Neuregulin-1 (NRG1), have been extensively studied for their role in interneuron tangential migration [33-35]. DISC1 has been hypothesized to converge with NRG1-ErbB4 cascades in modulating migration [28]. However, interneuron tangential migration deficits are likely to arise through the simultaneous dysregulation of not just one, but several protein interactions including cytoskeletal proteins [31], dysbindin [2], neurotrophins [36] and transcription factors [37-39]. Future research addressing how DISC1 can affect these various pathways will help to elucidate the precise molecular mechanisms by which DISC1 affects interneuron tangential migration.

Next we examined the number and positioning of interneurons in adult *Disc1*-L100P mice and found changes consistent with human post-mortem schizophrenia studies including reductions in PV immunoreactivity and abnormal laminar distribution patterns [4,8,30]. Interestingly, other mutant *Disc1* mouse models exhibit similar reductions of PV-interneurons in the PFC and aberrant cortical positioning [21-23]. This suggests that DISC1 protein disruptions may overlap among these different mouse models, with a common effect on interneuron genesis and incorporation of PV-interneurons into proper cortical layers. Interneuron genesis in the ganglionic eminence is likely to be controlled by different transcription factors [40], but the relationship between DISC1 and interneuron production remains to be determined.

Another theory is based on the pyramidal interneuron network gamma (PING) model, which suggests that PV-interneurons are recruited by glutamatergic inputs from pyramidal neurons [41]. Previously, misplaced cortical pyramidal neurons and reduced spine densities within layers III and V pyramidal neurons were found in the *Disc1*-L100P mutants [42]. Consequently, incorrect guidance cues and weakened excitatory drive may lead to less recruitment of PV-interneurons and aberrant cortical lamination [43].

Interestingly, the increase in CB immunoreactivity within the DLFC and PV-immunoreactivity within the CA1 and CA2/3 subfields of the hippocampus did not parallel those observed in post-mortem schizophrenia studies [44-46] and a truncated *Disc1* mouse model [22]. Despite the inconsistent findings in the literature, an increase in CB mRNA expression and immunoreactivity in the PFC has been reported in several post-mortem studies [47,48]. Compared to PV subpopulations, CB-interneurons are less extensively studied and thus their features in schizophrenia remain unclear. CB-interneurons may affect pyramidal neuron activity in a different way than PV-interneurons, since the two interneuron types have different electrophysiological and synaptic characteristics [6]. Furthermore, the increase in CB-interneurons may be a compensatory response to PV-interneuron reductions [47]. Moreover, DISC1 can have differential regional effects between the cortex and hippocampus, as evident from opposing neuronal migration and outgrowth effects in previous DISC1 knockdown studies [49,50]. Multiple pathways are likely to be involved in determining interneuron fate. Further research is required to elucidate the precise relationship between DISC1-related pathways and to understand the specific roles of DISC1 in different interneuron subpopulations.

As mentioned previously, reduced GAD67 expression in PV-expressing cells has been consistently reported in post-mortem brains of schizophrenia patients [7,8]. Here, we provide novel evidence of diminished GAD67/PV co-localization in *Disc1*-L100P mutants when compared to WT controls. Immunohistochemical analyses have confirmed the co-expression of DISC1 and GAD67 in GABAergic interneurons [51]. The *Disc1* L100P mutation may affect specific downstream transcription control of GAD67 enzyme levels, or GAD67 reduction may be a compensatory response to reduced PV immunoreactivity. Furthermore, western blots of GAD67 can provide information on whether GAD67 protein levels are changed in our *Disc1* mutants. The causes and functional relationship between DISC1 and GAD67 remain to be determined. Our findings provide a starting point for future research to elucidate the role of DISC1 in GABAergic signaling.

As mentioned previously, the immunoreactivity and distribution patterns of PV-immunostained cells have been extensively studied in human post-mortem and animal studies. However, the histological relationship between different interneuron subpopulations has not been examined. Given that our *Disc1*-L100P mutants displayed selective alterations in density and distribution of both PV- and CB-immunostained cells, future double interneuron immunolabeling experiments would provide important insights about whether the density and distribution of one interneuron subtype is associated with the other.

In conclusion, the results presented in this study support the notion that DISC1 plays a role in interneuron development. But whether DISC1 mutations are a primary cause of aberrant interneuron development through direct disruption of interactions with relevant proteins and transcription factors or produce secondary effects from disturbed pyramidal neuron positioning, remains to be determined. Moreover, investigating electrophysiological properties of the *Disc1*-L100P mouse cortex and the hippocampus [52] would be useful in addressing functional outcomes of these histological abnormalities. Nonetheless, we have provided an overview of interneuron histology and development in an *N*-ethyl-*N*-nitrosourea (*ENU*)-induced *Disc1*-L100P mouse line, which supplements our previous work in characterizing cortical abnormalities of pyramidal neurons [42]. Our findings further support the role of DISC1 in interneuron development and provide additional insights about how *Disc1* mutations can lead to the brain and cognitive abnormalities associated with schizophrenia. More importantly, this study represents a starting point for further investigation of DISC1-related mechanistic pathways in interneuron development.

## Methods

### Mice

*N*-ethyl-*N*-nitrosourea (*ENU*)-mutagenized *Disc1*-L100P homozygous mutant embryonic and adult mice (8 weeks) on a C57BL/6 background were generated as previously described [26]. WT littermates from the same breeding batch were used as controls. All mouse protocols were approved by the Centre for Addiction and Mental Health Animal Care Committee.

### Immunohistochemistry

Adult mice were sacrificed by cervical dislocation. Both embryonic and adult mouse brains were dissected, fixed in 4% paraformaldehyde, cryoprotected in 30% sucrose and stored at −80°C before further processing. Frozen coronal sections of 10 μm-thickness were cut using a cryostat (Bright Instrument Co. 5030). All sections were initially incubated in blocking solution (0.1M PBS, 1% Triton X-100, 0.5% Tween 20, 5% skim milk) for 2 hours at room temperature to reduce nonspecific background, followed by primary antibodies overnight at 4°C and secondary antibodies for 2 hours at room temperature. The following primary antibodies were used: anti-parvalbumin (1:200; Sigma-Aldrich), anti-calbindin D-28k (1:200; Millipore) and anti-GAD67 (1:100; Millipore). Fluorescent secondary antibodies conjugated to either Alexa 488 or 594 (1:200; Invitrogen) were used for detection of primary antibodies.

### Analysis of immunohistochemistry: interneuron densities and distribution

All immunohistochemical images were captured using a confocal microscope (Zeiss LSM510 Meta) at 10× magnification, converted to grey-scale and normalized to background staining. Sections chosen for analysis were anatomically-matched between comparing groups and included samples from rostral, medial and caudal regions. A two-dimensional cell counting approach was employed, with random sampling from fixed regions of interest (ROI) to provide accurate estimates of cell densities [53]. Fluorescent cells within each ROI were counted using the ITCN plugin for ImageJ (http://rsb.info.nih.gov/ij/) (ITCN parameters: width, 20–25 pixels; minimum distance, 10–13 pixels; threshold, 0.3 pixels) [42,54]. Anatomical regions were defined according to the Golgi Atlas of the Postnatal Mouse Brain [55]. Specific procedures for defining areas of analysis are described below.

### Interneuron density

A fixed rectangular ROI was positioned over the mPFC (1 mm high × 500 μm wide) and the DLFC (750 μm high × 1.6 mm) (Figure 2A). Similarly for the hippocampus, fixed areas were placed on the dentate gyrus (DG) (300 μm × 600 μm), CA1 and CA2/3 (400 μm × 400 μm) subfields of the hippocampus for analysis of interneuron subtype densities (Figure 6A). Only PV-labeled cells were counted in the DG as CB-interneurons were absent. Since CB also labels pyramidal neurons within the hippocampus, CB-labeled interneurons were distinguished and identified on the basis of their location outside the *stratum pyramidale* cell layer [56,57].

### Interneuron distribution

In the embryonic E14 and E16 brains, a selected curved region (300 μm wide) from the dorsal cortex to ventral preoptic area was outlined, straightened and divided into seven equal ROIs to capture the tangential migratory paths of newborn interneurons (ImageJ) (Figure 1A).

Analysis of both laminar and tangential interneuron distribution was performed only in adult brains. Four rectangular ROIs (laminar: fixed width of 800 μm but variable length spanning the thickness of the cortex; tangential: 1 mm high × 800 μm wide) were delineated across the neocortex with the long axis perpendicular to the pial surface (Figure 4A). Specifically for laminar distribution analyses, each ROI was further sub-divided into eight equal regions from the pia mater to the bottom edge of layer VI (Figure 3A). For all distribution measurements, the number of fluorescently-labeled cells in each bin was counted and expressed as a percentage of the total number within all bins. A percentage rather than absolute counts was used since the exact area covered by the ROI may differ for each brain section, and our objective was to ascertain a shift in distribution across the ROI.

### GAD67 and PV colocalization

Fluorescent images for GAD67/PV analysis were taken at 25× magnification. Three fixed square ROIs (350 × 350 μm^2^) were positioned over each of the two hemispheres across the neocortex. All images were blinded prior to analysis. Co-localization was defined by the experimenter as any overlap in staining. Both the number of GAD67^+^PV^+^ and total PV^+^ cells were counted manually and expressed as a percentage.

### Statistical analysis

Statistical differences between WT and *Disc1*-L100P mutants were analyzed using the Student’s two-tailed *t*-test or two-way ANOVA (SPSS 13.0), followed by Bonferroni’s correction for multiple testing. Data are expressed as mean ± standard error of mean (SEM). A significance level of *p* < 0.05 was used for all analyses.

## Abbreviations

CB: Calbindin; DISC1: Disrupted-in-Schizophrenia-1; DLFC: Dorsolateral frontal cortex; GAD67: Glutamic acid decarboxylase 67; mPFC: Medial prefrontal cortex; PV: Parvalbumin.

## Competing interests

All authors declare that they have no competing financial interests.

## Authors’ contributions

Albert Wong, Sabine Cordes, John Roder and Frankie Lee designed the study. Frankie Lee and Clement Zai performed all experiments and statistical analysis. Frankie Lee and Albert Wong prepared the first draft of the manuscript. All authors contributed to and have approved the final manuscript.

## Supplementary Material

Additional file 1: Figure S1 A decrease in average total number of PV- and CB-interneurons within the mPFC in *Disc1*-L100P adult mutants. *Disc1*-L100P mutants had significantly fewer average total PV- and CB-immunostained cells within the mPFC (left) but no significant difference within the DLFC (right) when compared to WT (n = 16 from 4 mice per group; *t*-test, *p* < 0.05). All data are shown as mean ± SEM; * *p* < 0.05 versus WT. CB, calbindin; DLFC, dorsal lateral frontal cortex; mPFC, medial prefrontal cortex; PV, parvalbumin. Click here for file
