# Supplementary figures and images for: Abnormal interneuron development in disrupted-in-schizophrenia-1 L100P mutant mice
Source: Mol Brain. 2013 Apr 30;6:20. doi: 10.1186/1756-6606-6-20 (PMC3648430; doi:10.1186/1756-6606-6-20)

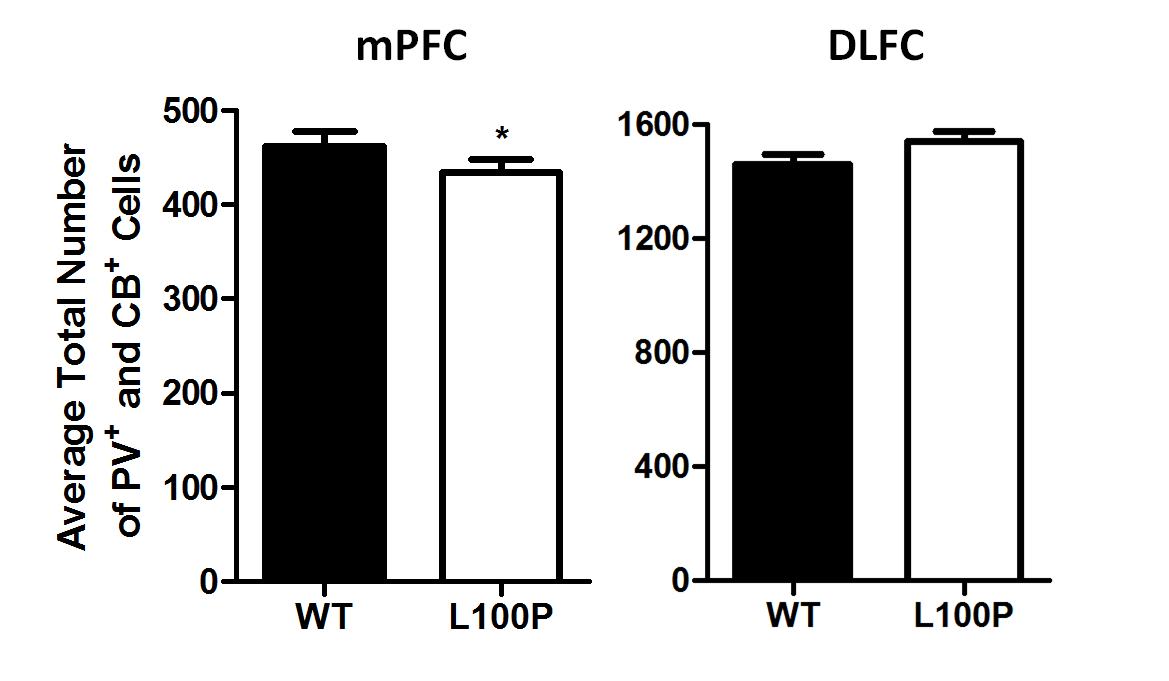

Supplement: Additional file 1: Figure S1 — A decrease in average total number of PV- and CB-interneurons within the mPFC in Disc1-L100P adult mutants. Disc1-L100P mutants had significantly fewer average total PV- and CB-immunostained cells within the mPFC (left) but no significant difference within the DLFC (right) when compared to WT (n = 16 from 4 mice per group; t-test, p < 0.05). All data are shown as mean ± SEM; * p < 0.05 versus WT. CB, calbindin; DLFC, dorsal lateral frontal cortex; mPFC, medial prefrontal cortex; PV, parvalbumin. [file 1756-6606-6-20-S1.tiff]
